# Process-Related Changes in Polyetherimide Joined by Friction-Based Injection Clinching Joining (F-ICJ)

**DOI:** 10.3390/ma13051027

**Published:** 2020-02-25

**Authors:** André B. Abibe, Marilia Sônego, Leonardo B. Canto, Jorge F. dos Santos, Sergio T. Amancio-Filho

**Affiliations:** 1 Helmholtz-Zentrum Geesthacht, Centre for Materials and Coastal Research, Institute of Materials Research, Materials Mechanics, Solid State Joining Process, 21502 Geesthacht, Germany; andre.abibe@gmail.com (A.B.A.); jorge.dos.santos@hzg.de (J.F.d.S.); 2 Graduate Program in Materials Science and Engineering (PPGCEM), Federal University of São Carlos (UFSCar), São Carlos 13.565-905, SP, Brazil; mrl.sonego@gmail.com (M.S.); leonardo@ufscar.br (L.B.C.); 3 Graz University of Technology, Institute of Materials Science, Joining and Forming, BMVIT Endowed Professorship for Aviation, 8010 Graz, Austria

**Keywords:** staking, hybrid structures, microstructural change, amorphous polymer, joining

## Abstract

This work presents a comprehensive study on the effects of the Friction-based Injection Clinching Joining (F-ICJ) process on the microstructure and local properties of the stake head. The manuscript evaluates the consequences on the quasi-static mechanical performance of hybrid joints of amorphous polyetherimide (PEI) with aluminium AA6082. Through an overlay of microhardness map on a cross-polarized transmitted-light optical microscopy (CP-TLOM) image, two lower-strength microstructural zones in the PEI stake head were observed: a plastically-deformed zone (PDZ) and a thermo-mechanically-affected zone (PTMAZ). When compared to the base material, PDZ and PTMAZ have a reduction of 12%–16% and 8%–12%, respectively, in local mechanical properties. The reduced local strength was associated with distinct volumes of loosely packed PEI chains with unsteady chain conformation and thus larger free volume in the affected regions. The mechanical strength reduction is reversible through physical aging by thermal annealing the joints, which additionally shows that process-induced thermomechanical degradation of PEI by chain scission, as evidenced by size exclusion chromatography (SEC) analysis, does not appear to affect local mechanical strength. An evaluation of typical loading regimes of staked joints in lap shear (average ultimate force of 1419 ± 43 N) and cross tensile (average ultimate force of 430 ± 44 N) testing indicates that the process-induced changes of PEI do not compromise the global mechanical performance of such a structure. These findings provide a better understanding of the relationships between processing, microstructure, and properties for further F-ICJ process optimization.

## 1. Introduction

Lightweight design has been established as one of the most successful strategies for the reduction of emissions in transport industry. By applying the right material in the right place, it is possible to obtain a multi-material structure with optimized weight and strength. This approach has driven research on several new joining methods which are potentially able to assemble these advanced polymer-metal hybrid structures. 

Several staking processes have been developed in recent years. Threaded Hole Friction Spot Welding (THFSW), based on filling of the pre-threaded metallic hole by melting and re-solidifying polymer, was used to join AA5052 aluminum to short-carbon-fiber-reinforced polypropylene (PP-SCF) composite [1]. The increase of the polymeric melted surface was due to higher rotational speeds, and therefore, the growth of loading bearing area resulted in higher strength and fracture energy of the joints. Another technique, named friction filling staking joining (FFSJ), was used to join aluminum and polypropylene sheets by filling a metallic hole with polymer to create a local stake [2]. The joining mechanism of FFSJ involves the mechanical interlocking of the formed stake and the partial adhesion of polymer-metal and polymer-polymer interfaces. FFSJ lap shear joints achieved maximum tensile shear strength of 13 MPa, which is comparable to state-of-art staking performance. Hahn and Finkeldey [3] used ultrasonic riveting and hot-air-sticking to join fiber-reinforced thermoplastics to steel. They proved that hot-air-sticking preserved the fibers better than ultrasonic riveting, resulting in a load-bearing performance 50%–90% higher.

However, the application of new technologies in demanding industries such as aircraft and automotive requires a deep comprehension of several aspects of the joints. Manufacturing processes often causes alterations to the microstructure of materials, which in turn affects the final properties and performance of the joint. Therefore, not only the mechanical performance and damage tolerance are important investigation subjects, but also the effects of the process on local material properties. This understanding is essential for clarifying the influence of the process on joint properties to target joint efficiency.

Although much of the research in this topic has been performed with metallic materials, there is interest on the behavior of polymer and polymer composites affected by various joining processes. Simōes and Rodrigues [4] used transmitted-light optical microscopy (TLOM) on thin samples of polymethymethacrylate (PMMA) friction-stir-welded (FSW) joints to identify its microstructural zones. TLOM with crossed polarizers (CP-TLOM) has been used to analyze residual stresses through photoelasticity in joints with transparent polymers. With CP-TLOM, Kiss and Czigány [5] observed a heat-affected zone (HAZ) defined by molecular orientation and residual stresses in FSW joints of poly-ethylene-terephtalate-glycol (PETG). In a similar manner, Krishnan et al. [6] analyzed the flow-induced residual fields in polycarbonate (PC) welded by ultrasonic, hot plate, and vibration welding. These examples are not only useful for the prediction of the joint’s behavior in service, but also to improve process development of new technologies.

The effect of these microstructural changes on local properties of the joined materials can be further investigated by indentation testing (microhardness) [2] and physical–chemical analyses. Indentation testing has been proven as a powerful method to identify structural changes in polymers [7]. The measured strength can be related to internal packing of polymeric chains (i.e., free volume) [8,9], whereas features of the indentations indicate inelastic and elastic contributions of deformation [7,10,11]. A number of analytical methods such as differential scanning calorimetry (DSC) [12,13], thermogravimetry (TGA) [14], and size exclusion chromatography (SEC) [15,16] have been used to identify physical–chemical changes in microstructural zones of joined materials. These combined analyses provide useful insights of the relationships between process control, microstructural changes, and local properties within the joint area.

This work describes the process-related changes observed in an amorphous engineering thermoplastic (polyetherimide; PEI) when joined to a metal (aluminum AA6082) by a friction-based staking process (F-ICJ [17]). The nature of the process-related changes is investigated by microstructural analyses, local mechanical properties, and physical–chemical properties of the polymer stake head. It is showed that PEI joined by F-ICJ presents a plastically deformed zone (PDZ) and a thermo-mechanically-affected zone (PTMAZ) of lower mechanical strength due to more loosely packed chains that increases the free volume in these regions. Although polymeric thermomechanical degradation takes place at some processing conditions, it does not seem to affect the mechanical properties of the joints as the local mechanical properties could be enhanced by annealing.

## 2. Friction-Based Injection Clinching Joining (F-ICJ)

**Figure 1:**
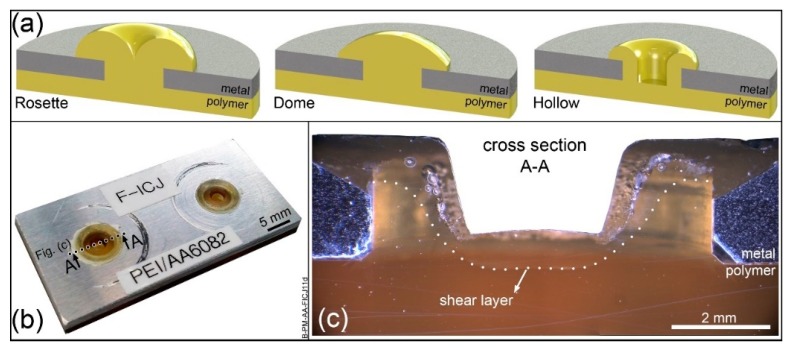
(**a**) Conventional stake designs for metal-polymer structures; (**b**) surface view of an F-ICJ structure; (**c**) cross-sectional view of an F-ICJ stake. Adapted from [19].

Friction-based Injection Clinching Joining (F-ICJ) has been recently explored as an alternative advanced staking process for new lightweight structures by Abibe et al. [17,18,19]. Typical staked structures use joints in the most common configurations of rosette, dome, or hollow stakes (Figure 1a) [20]. They provide a reliable process for simply attaching dissimilar materials. The strength of these joints comes from the large stake head, which can be a limitation in exterior or lightweight applications. 

F-ICJ polymer stake heads are flush to the surface of the metal part (Figure 1b). A thermomechanical process induces polymer flow within a shear layer to form the stake. The mechanical strength of an F-ICJ joint comes from anchoring of the stake in cavities inside of the through hole, made possible by the material flow in the shear layer (Figure 1c). This feature allows F-ICJ stakes to be smaller, lighter, and more aesthetically flexible than standard staking processes [17].

**Figure 2:**
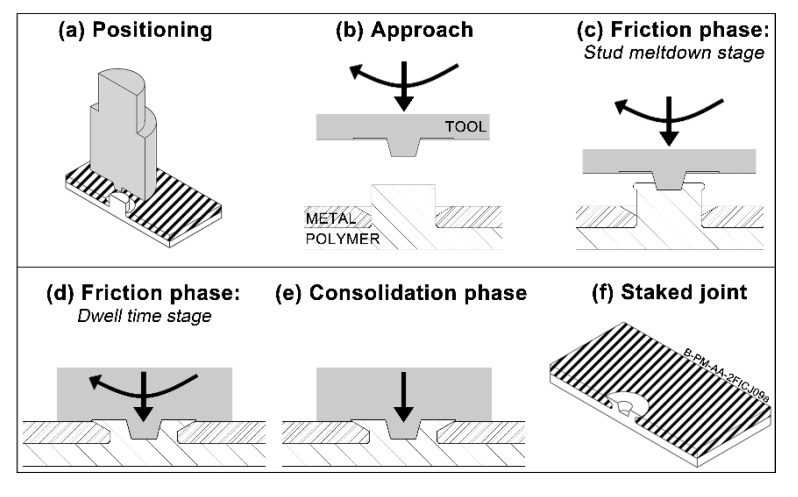
(**a**–**f**) Steps of the Friction-based Injection Clinching Joining (F-ICJ) process. Adapted from [18].

The basic process steps for the F-ICJ welding technique are shown in Figure 2. The thermoplastic and other joining components are pre-assembled (preferably on a backing plate) and aligned with the moving axis of the non-consumable tool (Figure 2a). After this positioning step, the rotating tool moves towards the thermoplastic stud (Figure 2b). The contact between the rotating tool and the stud generates frictional heat at their interface, gradually softening or melting the polymer, and allowing the tool to penetrate further into the stud (Figure 2c). The friction heats and deforms the thermoplastic stud, causing softening (or melting) and flow. Next, tool rotation stops and axial pressure acts further upon the molten polymer, pushing it into the cavities and shaping the final stake geometry (Figure 2d). The tool remains in this position until the thermoplastic is cooled (Figure 2e). The tool retreats and the F-ICJ joint is created.

The fundamentals of the F-ICJ process have been described in [18,19]. Joints are formed by providing frictional heat to a polymeric stud, which flows within a shear layer around the tool to create a stake. The stake is cooled down under pressure, avoiding large dimensional recovery. A stop-action procedure with monitoring of polymer temperature and process-related signals provide insight in the joint formation mechanisms. Material flow within the shear layer is fundamental for efficient filling of the cavities by the molten polymer, while also eliminating volumetric flaws. The preliminary investigation on the mechanical behavior identified the main failure mechanisms of joints in lap-shear and cross-tensile configurations. The benchmark study showed that F-ICJ is comparable to state-of-the-art ultrasonic staking in terms of mechanical properties, but needs improvement in cycle time.

## 3. Materials and Methods

**Figure 3:**
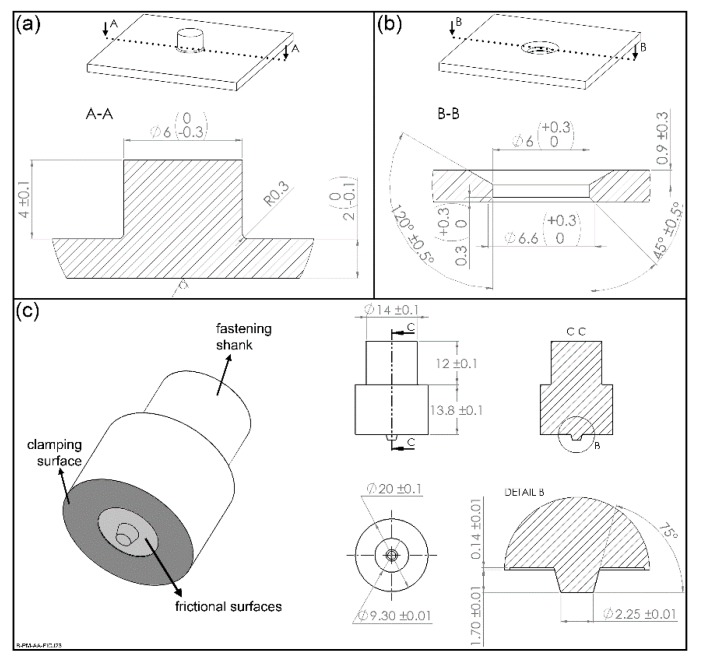
Base materials used. (**a**) Polyetherimide (PEI) part. (**b**) Aluminum 6082-T6 part; (**c**) F-ICJ tool made of stainless steel 316L.

Polymer parts with a stud were machined from 6.35 mm thick extruded polyetherimide plates (PEI, grade Duratron U1000 PEI, Quadrant Plastics, Lenzburg, Switzerland), as showed schematically in Figure 3a. The stud base has a radius of 0.3 mm to decrease stress concentration in this region. Through holes with a chamfer cavity were machined in 2 mm thick aluminum 6082-T6 plates (AA6082, Aalco Metals Ltd, Halesowen, UK), which fit the stud of the PEI part (Figure 3b). Although other specimen manufacturing methods can result in better final properties [21,22,23], conventional machining is still the most common technique adopted, which were selected here to simulate real conditions. A non-consumable tool of stainless steel 316L depicted in Figure 2c was used for the F-ICJ process.

The pre-assembled parts were joined by F-ICJ using an automated gantry system (model RNA, H.Loitz-Robotik, Hamburg, Germany) equipped with a high-speed friction welding machine (model RSM410, Harms + Wende, Hamburg, Germany). The system operates with rotational speeds ranging from 6000 to 21,000 rpm and axial forces of up to 24 kN. A torque sensor (model 9049, Kistler, Winterthur, Switzerland) was used to obtain the materials’ torque response. The unified system allows signal monitoring from rotational speed, axial force, spindle displacement, and torque. The specimens were cleaned with pressurized air (PEI) and acetone (AA6082) prior to joining. They were clamped in a standard sample holder to avoid slippage during joining. 

Microstructural analysis of PEI was carried out with a Leica DM IRM optical microscope (Leica Microsystems, Wetzlar, Germany). Reflected light optical microscopy (RLOM) and transmitted light optical microscopy (TLOM) were used for general microstructural and material flow observation. Samples were prepared by cutting the specimens 1 mm from their center and embedding them in low-temperature epoxy resin. Embedded samples were ground and polished for RLOM analyses according to the standard materiallographic procedure. For TLOM analyses, thin sections of 1 mm thickness were cut from embedded samples and subsequently had both sides polished.

**Figure 4:**
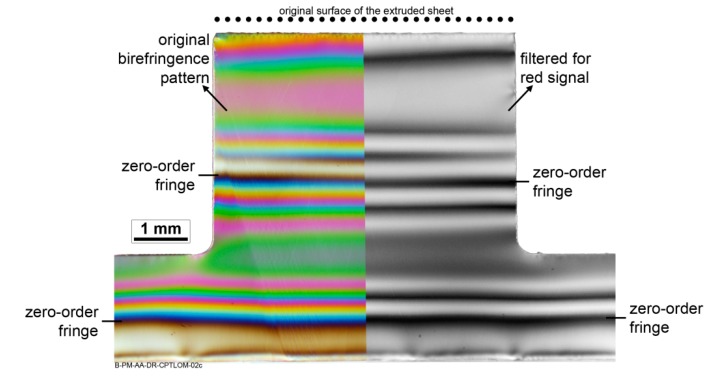
Transmitted-light optical microscopy with crossed polarizers (CP-TLOM) of the PEI base material. Left-hand image is the original output; right-hand image is filtered for red signal only. Adapted from [19].

Qualitative evaluation of residual stresses in the PEI part after joining was performed using transmitted optical microscopy with crossed polarizers (CP-TLOM) on 1 mm thick section samples. The light source was a standard microscope filament lamp producing a continuous white light spectrum. The observed image produces a colored fringe pattern, in which each isochromatic corresponds to a local stress level caused by F-ICJ. A grayscale image of this pattern can qualitatively indicate the stress levels. In this study, the zero-order fringes were identified in color images as the non-stressed regions, then the grayscale images were used to interpret the local stresses and its dependence on the F-ICJ process parameters. Typical colored and grayscale fringe patterns and zero-order fringe identification of the PEI base material are showed in Figure 4.

Local mechanical properties of PEI were measured on embedded and polished cross sections of joints. Zwick ZHV (Zwick Roell, Ulm, Germany) equipment was used with an indentation load of 0.495 N over 15 s, and distance between indentations of 200 µm. The testing procedures are based on the ASTM E384 [24].

Changes in molecular weight distribution (MWD) of PEI were evaluated by size exclusion chromatography (SEC) with a HT-GPC equipment (Viscotek, Berkshire, UK) using HT-806 M columns coupled to a refractive index detector. Samples of PEI were removed with a scalpel from the PTMAZ of each specimen, and dissolved in trichlorobenzene (TCB) in a heated bath at 150 °C for 10 min at concentration of 2 mg L^−1^. The analyses were performed using 200 µL of PEI/TCB solution at 150 °C and flow rate of 1 mL min^−1^. The calibration curve was built using monodisperse polystyrene standards with molecular weights between 845 and 1,900,000 g mol^−1^.

The energy input provided by each set of F-ICJ process parameters was calculated to establish a correlation with physical–chemical changes in the PEI. Mechanical work is commonly used to estimate energy input in friction welding processes [25,26]. Equation (1)) calculates mechanical work as the energy input $E_{work}$ for the F-ICJ process. The frictional contribution $E_{f}$ can be described by the product of the average angular velocity $\bar{\omega }$ and the integral of the torque M over the frictional time FT. The deformational contribution $E_{d}$ is calculated by the product of the frictional force FF and the integral of the tool displacement rate υ over FT.

(1) $$E_{work}=\;E_{f}+E_{d}=\bar{\omega }\int_{t_{0}}^{FT}M\;dt+FF\int_{t_{0}}^{FT}\upsilon \;dt=\;M_{total}\bar{\omega }+FF\;\Delta x\;\left[J\right]$$

Both constants FF and $\bar{\omega }$ are calculated from the experimental curves. The tool displacement  Δx is the result of the integral of the tool displacement rate υ over time. Within the parameter sets used in this work, the deformational component FF Δx contributed with a maximum of 10 J to energy input, amounting to less than 1% of total energy input. For simplification, only the rotational component $\;M_{total}\bar{\omega }$ was used in this work. The total torque $M_{total}$ is experimentally obtained from the torque curves of the friction phase (stud meltdown and dwell time stages).

## 4. Results and Discussion

### 4.1. Overview of the Microstructure of the Polymeric Stud

**Figure 5:**
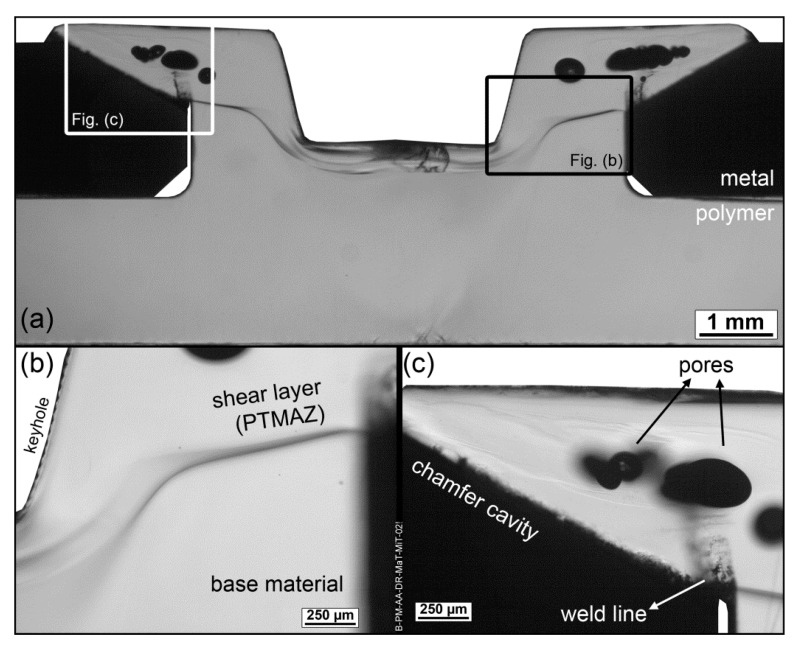
Microstructural features of an PEI-aluminum F-ICJ joint produced with a conical-pin tool and chamfer cavities. (**a**) Overview of joint cross-section; (**b**) polymer-polymer interface. (**c**) pores and remnant weld lines in the polymer thermo-mechanically-affected zone (PTMAZ; TLOM images). For processing conditions see Table 1. Adapted from [19].

A typical F-ICJ joint results from the effect of heating and deformation imposed by rotational and axial movement of the tool in contact with the polymeric stud. Its microstructure is highly influenced by heat input, which in turn depends on the tool geometry and process parameters. The cross-section of such a PEI-aluminum F-ICJ joint is shown in Figure 5, with details of its microstructural zones and joint features. This joint was produced with the set of parameters shown in Table 1.

The use of transmitted-light optical microscopy (TLOM) through a thin section of an F-ICJ joint makes it possible to observe the microstructural features and discontinuities in the polymer. A dark line across the diameter of the stake shaft delineates a polymer-polymer interface (Figure 5b). The volume above this interface interacted with the frictional surfaces of the conical-pin tool, and was heated and deformed by its rotation and axial force. This interface is the border of the shear layer displayed in Figure 1c. This is a polymer thermo-mechanically-affected zone (PTMAZ), which is characterized by material flow and the presence of volumetric discontinuities such as pores and remnant weld lines (Figure 5c).

**Figure 6:**
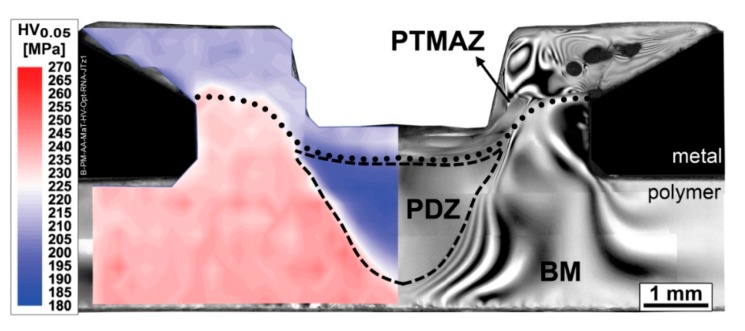
Overlay of a microhardness map (left) on a CP-TLOM micrograph of the PEI-aluminum F-ICJ joint from Figure 5. Dotted lines are the boundaries of the polymer thermo-mechanically-affected zone (PTMAZ); dashed lines are the boundaries of the plastically deformed zone (PDZ). Base material (BM) was labeled for the unaffected PEI region. For processing conditions see Table 1. Adapted from [19].

To better visualize the microstructural zones and understand their local properties, further characterization methods were performed. The right-hand side of Figure 6 presents a micrograph of the joint from Figure 5 by transmitted-light optical microscopy with crossed polarizers (CP-TLOM) that displays birefringence patterns. To complement the analysis and help to understand possible changes in the local mechanical properties of the polymer, a microhardness map of the joint produced with the same joining condition is overlaid on the left-hand side of Figure 6. The coupled analysis reveals three microstructural zones with different local mechanical properties: a polymer thermo-mechanically-affected zone (PTMAZ), a plastically deformed zone (PDZ), and unaffected base material (BM). At this resolution of the microhardness map (200 µm between indentations), no sharp transition zone between the PTMAZ and the BM can be identified that would otherwise characterize an extensive polymer heat-affected zone (PHAZ).

### 4.2. Microstructural Zones and Interfaces at the Polymeric Stud

#### 4.2.1. Plastically Deformed Zone (PDZ) and Base Material (BM)

Beneath the shear layer boundary of the PTMAZ (dotted line in Figure 6), two zones can be identified. The highest-strength volume BM has base material properties and is not affected by the process. The lowest-strength PDZ (boundaries marked by a dashed line in Figure 6) is directly below the conical pin’s line of action, and has 12%–16% less local strength compared to the base material. This region displays no signals of material flow as seen in the PTMAZ, indicating that the temperature of this volume is not significantly altered by the rotating action of the tool, so that this volume remains in a glassy state during processing. A well-formed PDZ is only observed for F-ICJ processing conditions where the axial joining force is notably high (above 2400 N) [19], creating stresses above the yielding point of the solid polymer; therefore it follows that the PDZ is plastically deformed by compression.

**Figure 7:**
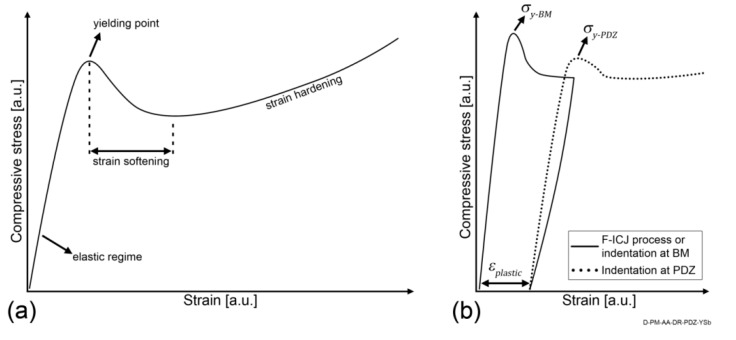
(**a**) Behavior of PEI under uniaxial compression (based on [27,32]). (**b**) Yielding of PEI up to strain softening (solid curve), and reloading the yielded PEI during microhardness (dotted curve). Adapted from [19].

PEI undergoes strain softening under compression in the 7%–13% strain range [27], as shown schematically in Figure 7a. Strain softening in polymer glasses is related to the difference in conditions (energy or stresses) required to initiate yielding and to propagate it [28]. In the case of the polymer in the PDZ, its mechanical history owing to F-ICJ can be schematically represented by the solid stress-strain curve in Figure 7b. The polymer in the PDZ is stressed up to a point in the strain softening region, and after removal of load a residual plastic strain $\varepsilon_{plastic}$ is present. When reloading a previously yielded amorphous polymer during the microhardness test (dotted curve in Figure 7b), a new lower yielding stress is reached ($\sigma_{y-PDZ}$), because the necessary conditions for initiation of yielding were previously achieved ($\sigma_{y-BM}$). A peak of yielding stress is usually still present, due to a certain level of physical aging (hardening as a result of the reduction of free volume at temperatures close to but below the glass-transition (T_g_)) during cooling or at room temperature. Plastic deformation by yielding is described as conformational changes of the chains, leading to increased free volume in amorphous polymers [28,29,30,31], which is detected as reduced hardness in indentation testing [8,9].

Identification of the shape and limits of the PDZ can be additionally supported by the birefringence pattern with crossed polarizers. Birefringence patterns are associated with the residual stresses in a material through the stress-optic law (Equation (2) [33]).

(2) $$\sigma_{res}=\left(\sigma_{1}-\sigma_{2}\right)=\left(n_{1}-n_{2}\right)\;C_{opt}=\frac{\delta }{y}C_{opt}\;\left[MPa\right]$$

where $\sigma_{res}$ is the residual stress, $\left(\sigma_{1}-\sigma_{2}\right)$ is the difference in normal stresses in the specimen, $\left(n_{1}-n_{2}\right)$ is the birefringence, $C_{opt}$ is the stress-optical coefficient of the material, δ is the retardation of light in the specimen, and y is the specimen thickness. Photoelasticity [34] can be used to quantify stresses in birefringence patterns by defining these parameters. However, a quantitative measurement of residual stresses through the photoelastic effect requires adequate equipment and complex analyses [35,36], which were not within the scope of this work. Such analyses can be performed more commonly using a color image and obtaining the intensity of the red–green–blue signals, or by using a Michel–Levy chart. These methods assist in the definition of the number of fringes in a certain region, which are a measurement of the retardation δ [37]. In this work a grayscale image filtered from the red signal was used to qualitatively estimate the number of fringes in a given region of the polymer joint. An increasing number of fringes (larger retardation δ) can be associated with an increasing level of residual stresses (see Equation (2)) [33].

**Figure 8:**
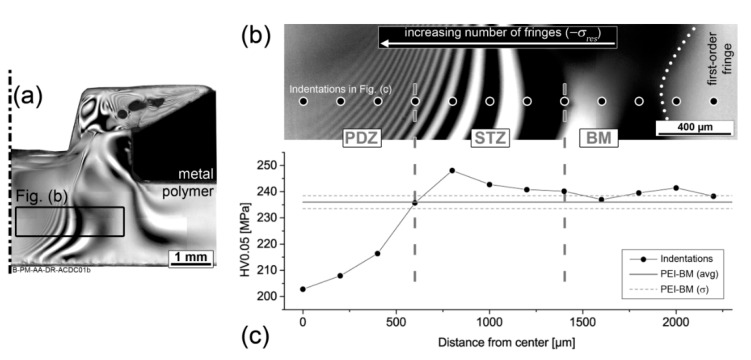
(**a**) Half of a cross-sectional view of a PEI-aluminum F-ICJ joint featuring a PDZ. (**b**) Birefringence of the PDZ-BM transition highlighted in (**a**), with number of fringes ($-\sigma_{res}$ ) increasing in the PDZ direction (CP-TLOM). (**c**) Indentation profile highlighted in (**b**), showing the formation of a strengthened transition zone (STZ) between the PDZ and BM. The gray horizontal lines in (**c**) correspond to the PEI base material’s hardness. For processing conditions see Table 1. Adapted from [19].

Figure 8a is one half of the cross-section previously presented in Figure 6; the transition region between the PDZ and BM zones is highlighted with a black rectangle. This highlighted region is shown in greater detail in Figure 8b. In this image, a first-order fringe is on the right-hand side, and increasing orders of isochromatic fringes can be observed towards the PDZ on the left of the figure. The brighter, well-defined fringes in the base material region are low-order fringes, indicating lower stresses, while the lighter shades in the PDZ are high-order fringes, associated with high residual stresses [35,38]. The high-order fringes correspond to higher retardation (δ) values, which indicate higher residual stresses, as described in Equation (2). Therefore, yielding in the PDZ creates a highly-stressed volume, whereas away from the PDZ and into the BM the fringe orders are of the same level as observed in the as-received material (Figure 4) where no significant residual stresses are present.

It can be also observed in Figure 8c that local mechanical strength—represented by microhardness values—is decreased at the PDZ. The black dots in Figure 8b represent the position of the indentations of the profile shown in Figure 8c, covering the transition between PDZ and BM. The horizontal gray lines in Figure 8c indicate the as-received hardness of PEI and its standard deviation, and the black disks are the indentation measurements of the profile. It is possible to see that a strengthened transition zone (STZ) was detected between the BM and PDZ. It is known that compressive stresses increase hardness values [10,11], whereas plastic deformation decrease them [7,8]. Therefore, there are two competing effects taking place in the STZ. The combined analysis demonstrates that in the PDZ and STZ significant compressive residual stresses ($-\sigma_{res}$) are present, but in the PDZ the effect of free volume increasing because of yielding dominates and hardness is lower, whereas in the STZ no yielding is present and the $-\sigma_{res}$ increases hardness. In the BM, none of these effects play a role and the hardness values are in the range of the as-received material.

#### 4.2.2. Polymer Thermo-Mechanically-Affected Zone (PTMAZ)

Following the polymer–polymer interface line in Figure 5b, the volume previously described as the PTMAZ presents 8%–12% lower strength (as shown in the hardness map of Figure 6) than the base material. The PTMAZ corresponds to the shear layer described in Figure 1c. This is a polymer volume that is affected by frictional heat and shear stresses, and changes in local properties are related to the thermomechanical processing and its subsequent thermal history in service. Unlike the PDZ, no effects of yielding below T_g_ are seen, because this volume is above the T_g_ of polymer during processing. Competing phenomena may act in this volume to change its microhardness: topological changes affecting internal order, and molecular weight changes [7]. Conformational chain changes that alter entanglement density occur from quenching (increasing free volume) or physical aging (reducing free volume). Lower hardness in this case indicates larger free volume [8,9,39]. Reduction of molecular weight by chain scission tends to reduce microhardness, because a larger fraction of chains with low molecular weight represent more chain ends and degradation products [40]. By contrast, thermally-induced crosslinking increases local strength as a result of a rigid network with lower free volume [7]. A combination of entanglement density and molecular weight reduction is probably associated with microhardness reduction in the PTMAZ.

Chain entanglement density in PEI at the stake head of F-ICJ joints is related to the available energy for chain diffusion at the end of the friction phase. During the friction phase, the PTMAZ reaches temperatures (up to 385 °C) far above the T_g_ of PEI (215 °C), and it cools down rapidly (≈35 °C·s^−1^) during consolidation [19]. At the end of the consolidation phase the polymer is well below T_g_ in a glassy state [33]; this does not allow the chains to achieve a densely packed conformation, and therefore reduces the local strength compared to the as-received polymer [9,32]. This indicates that the cooling regime in the PTMAZ is probably faster than for the manufacturing process of the as-received material. The extruded thick PEI sheets either use slow cooling after exiting the die, or are annealed after extrusion to achieve a relaxed chain conformation [41]. Either way the industrial process allows the polymer chains to achieve a more packed chain conformation than the as-joined PTMAZ. The local mechanical properties of PTMAZ are also lower than its base material due to physical–chemical effects in FFSJ joints [2].

### 4.3. Physical–Chemical Changes in the Microstructural Zones of F-ICJ Joints

**Figure 9:**
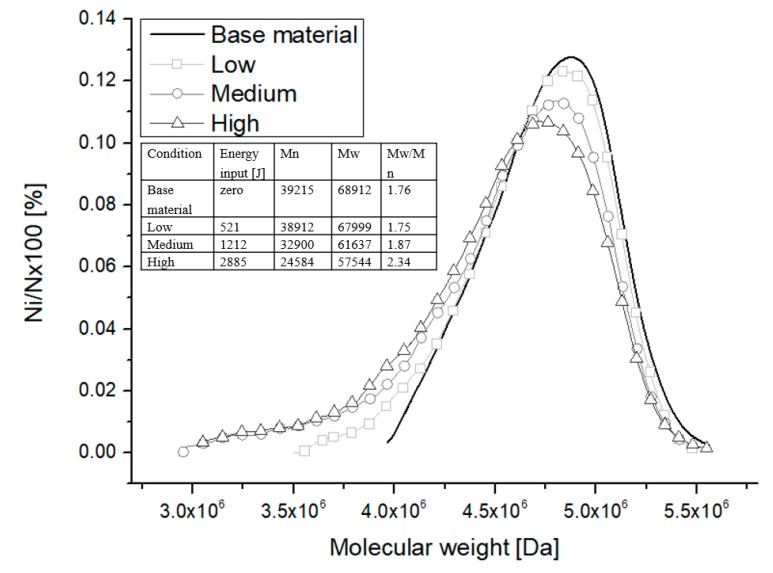
Molecular weight distribution curves of base material and PEI from F-ICJ joints at three levels of energy input: low ($E_{work}$ = 521 J), medium ($E_{work}$ = 1212 J), and high ($E_{work}$ = 2885 J) energy inputs. Adapted from [42].

To investigate if a certain level of degradation is present, physical–chemical properties related to chain length were studied. The hypothesis of PEI degradation caused by F-ICJ stated in the previous sections is proven in Figure 9 with measurements of molecular weight distribution (MWD) through SEC. The MWD for the base material is shown along with the MWD from the PTMAZ of joints with different levels of energy input $E_{work}$. A trend of lowering the average molecular weight (M_n_ and M_w_) and increasing of the polydispersity (M_w_/M_n_) towards higher energy input levels can be observed. This trend is an indication that the temperature and shear rate imposed by the F-ICJ process are high enough to cause thermomechanical degradation of PEI through chain scission [42]. Previous work by Sônego [42] showed that breakage of the imide and ether bonds cause multiple non-random chain scission, resulting in a considerable increase in the fraction of low-molecular-weight chains.

In the previous discussion, it has been shown that microstructural changes in PEI joined by F-ICJ decrease local strength in the PTMAZ and PDZ (Figure 6) and created residual stress gradients around the PDZ (Figure 8). To verify the assumption of increased free volume in the PDZ, and whether changes to the molecular weight also affect the local strength in the PTMAZ, a replicate of the high-energy-input joint from Figure 9 was subsequently annealed for 24 h at 200 °C (Tg—15 °C). The time and temperature of annealing were based on the maximized physical aging of PEI as reported by Belana et al. [43]. Annealing of amorphous polymers promotes accelerated physical aging. Through this annealing procedure the majority of free-volume effects on mechanical strength is removed [29,39], therefore making the effects of lower molecular weight on PEI joined by F-ICJ visible.

**Figure 10:**
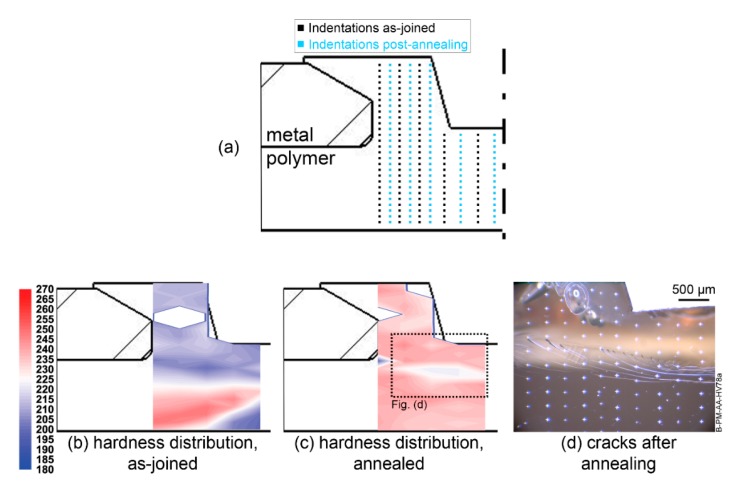
(**a**) Indentation positions for the annealing study; microhardness distribution maps from (**b**) as-joined and (**c**) annealed high-energy-input F-ICJ joint ($E_{work}$ = 2339 J). (**d**) Cracks in the polymer after annealing.

A cross-section of the joint showing the indentation positions prior to and after thermal aging are showed in Figure 10a. Five vertical profiles were executed before aging (black lines); and after aging five further profiles (blue lines) were indented between the previous ones. Local mechanical strength distribution is showed for the as-joined joint in Figure 10b and for the aged joint in Figure 10c.

The microhardness distribution for the as-joined PEI shows clear boundaries for the typical low-strength PTMAZ and PDZ of an F-ICJ joint. After annealing no microstructural zones can be distinguished. A region of lower strength is observed below the keyhole, corresponding to non-process-related cracks which develop during annealing (Figure 10d). Average measured microhardness was 235.0 ± 6.2 MPa across the annealed joint. The homogeneity of local mechanical properties over the joint indicates that any differences between the base material and PTMAZ or PDZ in the as-joined specimen were due to an unsteady chain conformation with increased free volume. Although thermomechanical degradation was present at the PTMAZ of this joint (high energy input, Figure 9), no noticeable difference in local strength can be measured after accelerated physical aging. Therefore, thermal annealing after F-ICJ process can enhance the local mechanical properties of joints, similar to ball-burnishing in the friction stir welding process [44,45].

### 4.4. Effect of the PTMAZ on the Joint Mechanical Behavior

It is of interest to correlate significant microstructural changes in a joint with the expected mechanical behavior of the structure in service. The mechanical behavior of staked joints is commonly tested through lap-shear and cross-tensile configurations. These tests simulate typical stresses in rivet-like assemblies.

**Figure 11:**
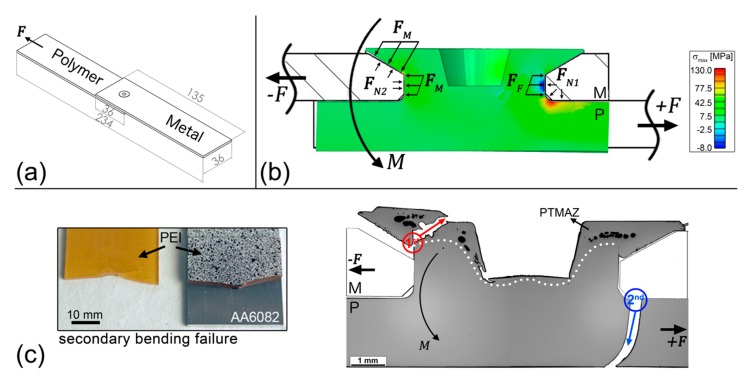
(**a**) Specimen and forces configuration for lap shear tests. (**b**) Stress concentration regions in an F-ICJ joint during lap shear tests. (**c**) Typical failure mode of F-ICJ lap shear specimens.

Lap shear tests are carried out in overlap specimens as depicted in Figure 11a. The lap shear joints had an average ultimate force of 1419 ± 43 N. In a metal-polymer configuration, the metallic part transfers the load to the stake shaft, practically shearing it from the polymer base plate. The distinct stiffness of the materials creates a secondary bending moment during this test, which generates the forces acting as drawn in Figure 11b. The secondary bending forces the rotation of the stake head as represented by the torque M. As a result, from M and F, the metal plate transfers load to the polymer in the form of $F_{M}$ and $F_{F}$. These forces, along with the reaction forces of the polymer part $F_{N1}$ and $F_{N2}$, create the stress field showed in Figure 11b, as seen by FEM simulation of F-ICJ PEI/AA6082-T6 joints. Compressive stresses are present on the stake shaft, while high-magnitude tensile stresses arise on the stake base. Lower-magnitude tensile stresses are present on the stake head at the opposite side, as a result from the secondary bending effect. The high tensile stresses on the stake base lead to a failure of the base plate as showed in Figure 11c. A detailed description of the failure mode was described in [19]. Summarizing, the load is supported at the marked regions both by the stake head (red circle) and the stake base (blue circle). The stake head contributes to diminish the secondary bending effect. As the secondary bending intensifies, the stake head stops supporting the load, and a main crack at the stake base grows rapidly leading to a catastrophic failure. 

A correlation can be established with the as-joined microstructure of an F-ICJ joint. At the stake base, where the final crack grows, there were no microstructural changes due to the joining process. This region’s resistance to failure can mainly be improved by geometrical design. On the other hand, the stake head region is the PTMAZ, with lower local mechanical strength. The stake head crack grows through this volume, and the crack growth is influenced by its properties and features. For instance, extreme levels of thermal–mechanical degradation may reduce PTMAZ strength to a point where the crack growth is facilitated. The presence of pores (Figure 5c) allows for a shorter crack growth path, accelerating the failure. Therefore, optimizing the microstructure and properties of the PTMAZ can increase the reliability of staked structures by halting the initial failure mechanisms.

**Figure 12:**
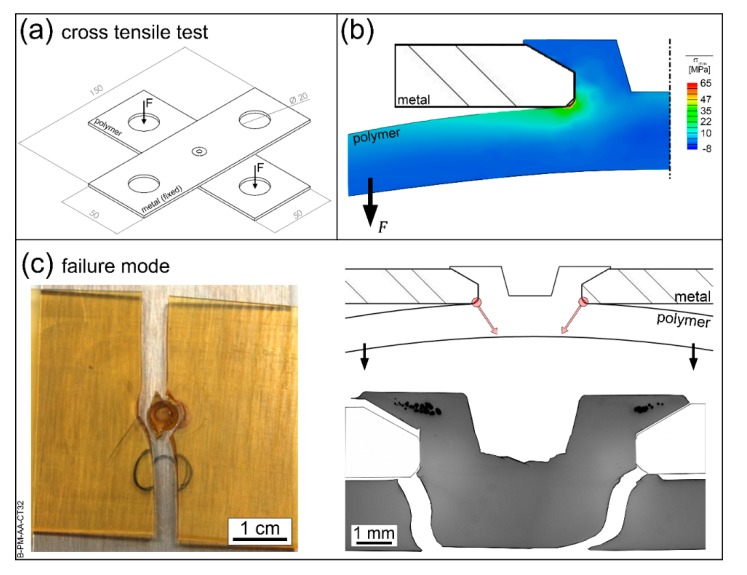
(**a**) Specimen and test configuration for cross tensile testing. (**b**) Stress concentration regions in an F-ICJ joint during cross tensile testing. (**c**) Main failure mode of F-ICJ cross tensile specimens.

Cross-tensile tests use an overlap specimen as shown schematically in Figure 12a. The joints had an average ultimate force of 430 ± 44 N in this test configuration. The metal plate is fixed, while the polymer plate is pulled away. This induces a tensile stress on the stake. Figure 12b shows the stress concentrations through FEM for a PEI-aluminum joint during cross tensile testing. Pulling down the polymeric plate creates a bending moment on the plate, which results in tensile stresses at the stake base. No significant stresses were observed at the stake head itself, showing that the PTMAZ is not bearing loads in such a configuration. Such a stress distribution leads most frequently to a failure by base-plate bending, where cracks nucleate at the stake base and grow rapidly towards the base plate’s lower surface (Figure 12c). The stake head is not damaged in such a failure mode. Differently from fastener staking [46], THFSW [1], and FFSJ [2] joints, the joining mechanism of F-ICJ does not depend on polymer-metal adhesion, as it relies mostly on the mechanical interlocking of the components. 

Typically, the lower strength and degradation of the PTMAZ will not influence the reliability of assemblies suffering loads as from cross tensile test. The base plate bending failure is a function of the base material’s properties and mechanical design of the polymeric part. However, in some cases foreign particles in the PTMAZ have been shown to nucleate cracks at the stake head during cross tensile tests [19]. The growth of these cracks is facilitated by the presence of pores, whose relative volume in the stake head increases with increasing energy input [19]. Therefore, it is generally advantageous to optimize the process using reduced energy input and a clean production. As shown in Figure 10 and Figure 11, the main crack in F-ICJ joints propagates through the polymeric base plate. Such behavior was also found in FFSJ joints [2]. Although the stud base was machined with a radius of 0.3 mm to avoid stress concentration, other geometries may have a better effect on the joint failure mode and should be investigated in the future.

## 5. Conclusions

- The joining of polyetherimide (PEI) amorphous engineering thermoplastic to aluminum alloy through the new F-ICJ staking joining process was presented. A comprehensive study on the effects of processing on the microstructure and local properties was carried out through light optical microscopy, microhardness testing, and size exclusion chromatography techniques.
- An analysis through qualitative transmitted-light optical microscopy combined with quantitative microhardness testing allowed to identify and clearly delimitate two microstructural zones in the stake head of PEI: a thermo-mechanically-affected zone (PTMAZ) and a plastically-deformed zone (PDZ).
- The PTMAZ, a polymer layer below the keyhole that was molten and sheared by the action the stirring tool at temperatures up to 385 °C well above the T_g_ of PEI (215 °C), and quickly cooled (≈35 °C s^−1^) afterwards, presented an 8%–12% reduction in the microhardness values compared to the base material (BM), as well as a few volumetric defects. This zone was characterized by a distinct birefringence pattern, as revealed by cross-polarized transmitted-light optical microscopy (CP-TLOM) analysis, resulting from thermomechanically-induced residual stresses. Furthermore, thermomechanical degradation of PEI by chain scission was identified through size exclusion chromatography (SEC) analysis.
- The PDZ, a polymer volume beneath the PTMAZ boundary that underwent strain softening as a consequence of developed compressive stresses resulting from F-ICJ, showed a 12%–16% reduction in the microhardness values and a different birefringence pattern. The boundary between the PDZ and the base material (BM) was characterized by the difference in the number of fringes presented in the CP-TLOM image.
- A post-joining annealing treatment eliminated residual stresses in the PTMAZ and PDZ, as a consequence of physical ageing of PEI. This helped to identify the nature of the above-mentioned microstructural local changes as distinct volumes of loosely packed PEI chains with unsteady chain conformation and thus larger free volume, which in turn reduced microhardness values. Although thermomechanical degradation of PEI on the staked head was evidenced by SEC, it seems not to contribute to the reduction in joint global mechanical strength.
- The consequences of the microstructural changes and thermal degradation of PEI on the global mechanical properties of staked joints were evaluated in terms of typical mechanical loading in lap shear (average ultimate force of 1419 ± 43 N) and cross tensile (average ultimate force of 430 ± 44 N) testing. Neither of the loading situations rely largely on the PDZ and PTMAZ, therefore the process-induced local strength reduction and PEI degradation by chain scission in the stake head do not compromise global mechanical properties of staked PEI-aluminum joints.

These findings extend the understanding of the relationships between processing, microstructure, and properties, as well as provide the basis for further F-ICJ process optimization.

## Figures and Tables

**Table 1:** Parameters set used for Friction-based Injection Clinching Joining (F-ICJ) joint production. $E_{work}$= 1415 ± 7 J.

Phase	Duration [ms]	Rotational Speed [rpm]	Axial Force [N]
Stud meltdown	765	7472	2551
Dwell time	1812	7018	2551
Consolidation	5000	0	5363
